# The BLUP method in evaluation of breeding values of Russian spring wheat lines using micro- and macroelements in seeds

**DOI:** 10.18699/vjgb-24-51

**Published:** 2024-07

**Authors:** N.A. Potapova, A.S. Zlobin, I.N. Leonova, E.A. Salina, Y.A. Tsepilov

**Affiliations:** Kurchatov Genomic Center of ICG SB RAS, Novosibirsk, Russia Institute for Information Transmission Problems of the Russian Academy of Sciences (Kharkevich Institute), Moscow, Russia Lopukhin Federal Research and Clinical Center of Physical-Chemical Medicine of Federal Medical-Biological Agency, Moscow, Russia; Kurchatov Genomic Center of ICG SB RAS, Novosibirsk, Russia; Institute of Cytology and Genetics of the Siberian Branch of the Russian Academy of Sciences, Novosibirsk, Russia; Kurchatov Genomic Center of ICG SB RAS, Novosibirsk, Russia; Kurchatov Genomic Center of ICG SB RAS, Novosibirsk, Russia

**Keywords:** genomic selection, BLUP, wheat, microelements, macroelement, геномная селекция, BLUP, пшеница, микроэлементы, макроэлементы

## Abstract

Genomic selection is a technology that allows for the determination of the genetic value of varieties of agricultural plants and animal breeds, based on information about genotypes and phenotypes. The measured breeding value (BV) for varieties and breeds in relation to the target trait allows breeding stages to be thoroughly planned and the parent forms suitable for crossing to be chosen. In this work, the BLUP method was used to assess the breeding value of 149 Russian varieties and introgression lines (4 measurements for each variety or line, 596 phenotypic points) of spring wheat according to the content of seven chemical elements in the grain – K, Ca, Mg, Mn, Fe, Zn, Cu. The quality of the evaluation of breeding values was assessed using cross-validation, when the sample was randomly divided into five parts, one of which was chosen as a test population. The following average values of the Pearson correlation were obtained for predicting the concentration of trace elements: K – 0.67, Ca – 0.61, Mg – 0.4, Mn – 0.5, Fe – 0.38, Zn – 0.46, Cu – 0.48. Out of the 35 models studied, the p-value was below the nominal significant threshold (p-value < 0.05) for 28 models. For 11 models, the p-value was significant after correction for multiple testing (p-value < 0.001). For Ca and K, four out of five models and for Mn two out of five models had a p-value below the threshold adjusted for multiple testing. For 30 varieties that showed the best varietal values for Ca, K and Mn, the average breeding value was 296.43, 785.11 and 4.87 mg/kg higher, respectively, than the average breeding value of the population. The results obtained show the relevance of the application of genomic selection models even in such limited-size samples. The models for K, Ca and Mn are suitable for assessing the breeding value of Russian wheat varieties based on these characteristics.

## Abstract (ru)

Геномная селекция – это технология, позволяющая определять генетическую ценность сортов сельскохозяйственных растений и пород животных, опираясь на информацию о генотипах и фенотипах. Измеренная селекционная ценность по отношению к целевому признаку дает возможность грамотно планировать этапы селекции и выбирать подходящие для скрещивания родительские формы. В настоящей работе использован метод BLUP для оценки селекционной ценности 149 российских сортов и интрогрессивных линий (4 измерения для каждого сорта или линии, 596 фенотипических точек) яровой пшеницы по содержанию семи химических эле- ментов в зерне – K, Ca, Mg, Mn, Fe, Zn, Cu. Качество оценки селекционной ценности было определено с помощью кросc-валидации методом случайного разделения выборки на пять частей, одна из которых выступала в качестве тестовой популяции. Средние значения коэффициента корреляции Пирсона для предсказания концентрации микроэлементов составили: K – 0.67, Ca – 0.61, Mg – 0.4, Mn – 0.5, Fe – 0.38, Zn – 0.46, Cu – 0.48. Для 28 из 35 иссле- дуемых моделей значение p-value было ниже номинального значимого порога (p-value <0.05). Для 11 моделей p-value было значимо после коррекции на множественное тестирование (p-value < 0.001). Четыре из пяти моде- лей для Ca и K, и две из пяти для Mn имели p-value ниже порога, поправленного на множественное тестирование. Для 30 сортов, показавших лучшие значения сортовой ценности, средняя селекционная ценность для Ca, K и Mn была выше на 296.43, 785.11 и 4.87 мг/кг соответственно, чем средняя селекционная ценность популяции. Полу- ченные результаты демонстрируют возможность применения моделей геномной селекции на ограниченных по размеру выборках образцов. Модели для K, Ca и Mn, показавшие наилучший результат, пригодны для оценки селекционной ценности российских сортов пшеницы для данных признаков.

## Introduction

Since time immemorial, classical selection methods have been used to develop new varieties of agricultural plants and animal breeds based on the hybridization of samples with economically valuable traits, followed by selection based on phenotype. With the evolution of genome sequencing technologies and methods for developing molecular markers, it became possible to use differences in the structure of genomes, find marker-trait associations, and use the information obtained to identify the relationship between genotypic polymorphisms and phenotypic variations. New approaches actively being developed in plants include marker-assisted selection (MAS) and genomic selection (GS) (Charmet, Storlie, 2012; Bhat et al., 2016; Bartholomé et al., 2022; Miller et al., 2023); they are also used for animals (Kuznetsov, 1999; Melucci et al., 2009; Suslina et al., 2019; Stolpovsky et al., 2020; Zhumanov et al., 2021; Johnsson, 2023).

Despite the fact that MAS is quite effective for searching for and introducing genes with a high contribution to the phenotypic manifestation of a trait, the main disadvantage of the method is the insufficient accuracy of predicting traits with polygenic inheritance. As an alternative to MAS, and for the purpose of overcoming the limitations of this method, genomic selection has been proposed. One of its main advantages is the use of predictive models to assess the breeding value (BV).

Among the main approaches for genomic selection the following methods can be distinguished: BLUP methods (Best Linear Unbiased Prediction) (Charmet, Storlie, 2012; Hoffstetter et al., 2016; Lozada, Carter, 2020; Plavšin et al., 2022), a group of Bayesian methods (Juliana et al., 2022) and a group of methods that use machine learning (Wang et al., 2018). Index methods are also used (Lopez-Cruz et al., 2020). Genomic selection methods in agriculture can change and increase the accuracy of the approach to breeding new plant varieties and animal breeds

Significant benefits of the use of GS have been observed in livestock production due to the high cost of reproduction. The adoption of GS for crop production began much later, but the general potential of current approaches and the potential of GS itself has now been explored in major crops such as wheat, corn, barley and soybean

Wheat plays an important role in food security around the world. In addition to nutritional components, wheat grains contain elements such as calcium, zinc, magnesium, etc. Elemental deficiency, also known as “hidden hunger,” occurs as a result of consuming food with low concentrations of elements and vitamins (Liu et al., 2019) and can lead to various diseases and even death. For this reason, the ability to intelligently select wheat varieties and increase the concentration of essential elements in the grain is an important way to combat nutritional deficiencies around the world. According to the elements’ content in the human body, elements are divided into macroelements (the content in the human body is hundredths of a percent or more), microelements (the content is from hundred thousandths to thousandths of a percent) and ultramicroelements (millionths of a percent or less). Of the seven elements analyzed in this paper, macroelements include calcium, potassium and magnesium, and microelements include iron, manganese, copper and zinc.

Genomic selection methods are applied to different populations and varieties of wheat for a variety of traits: from grain element content and yield to disease resistance (Hoffstetter et al., 2016). The most actively used method is BLUP and its various modifications – rrBLUP, gBLUP, egBLUP, wBLUP and others (Zhao et al., 2014; Martini et al., 2017; Berkner et al., 2022; Rabieyan et al., 2022). This method has proven itself over several decades of use in plant and animal breeding. The Bayesian method and its modifications are also used – BRR (Bayesian Ridge Regression), BL (Bayesian Lasso), BA (Bayes A), BB (Bayes B), BC (Bayes C). Recently, machine learning and deep learning methods have been applied to genomic breeding of wheat (Sandhu et al., 2021a, b; Sirsat et al., 2022). Comparisons of results between methods showed that they mainly overlap and the generally accepted methods of the BLUP group are in no way inferior to Bayesian methods and machine learning methods (Tsai et al., 2020; Berkner et al., 2022; Juliana et al., 2022).

Previously, using a panel of varieties and introgressive lines of bread wheat, we performed associative mapping of genetic factors that determine the content of seven chemical elements in wheat grain (Potapova et al., 2023).

The purpose of this work was to study the BV of samples from this collection based on the content of chemical elements and to obtain unbiased estimates of the effects of genetic polymorphisms to determine the BV of other Russian varieties

## Materials and methods

A panel of 157 Russian varieties and introgressive lines of spring soft wheat was used in the work. A list of plant material, information about the origin of samples and phenotyping conditions are available in (Leonova et al., 2020; Potapova et al., 2023).

Genotyping of samples with SNP markers was performed using the Illumina Infinium 15 K platform (TraitGenetics Section, Germany, www.sgs-institut-fresenius.de). After alignment of markers to the reference wheat genome to determine their location (chromosome and position), quality control checks and subsequent data filtering (quality of SNP genotyping < 5 %, minor allele frequency < 1 %, quality of sample genotyping < 5 %), 149 wheat lines and 11,405 SNPs (single nucleotide polymorphisms) remained. Detailed information about the analysis of genotypes is available in (Potapova et al., 2023).

The content of micro- and macroelements (Zn, Mg, Mn, Ca, Cu, Fe and K) was determined by flame spray atomic absorption spectrometry on a Contra 800 D device (Analytik Jena, Germany), as described in (Potapova et al., 2023). Statistical processing of the results was carried out using the Statistica v.10.0 software package

The assessment of the chemical elements content was carried out using seed material from the collection grown under the conditions of 2018–2019. At the same time, 4 measurements were performed, 2 for each year. Heritability was calculated using the formula V(G)/V(P) in the plink program (v.1.90b6.26, Purcell et al., 2007)

For each of these phenotypes (element content in grain), average values were obtained among 4 measurement points. The obtained average values were used in further analysis.

**Fig. 1.**
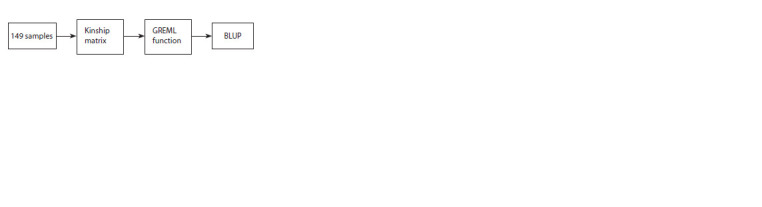
Analysis flowchart.

BLUP and cross-validation. Each element was analysed separately, analysis flowchart is presented in Figure 1.

For 149 cultivars, the genetic relationship matrix for SNPs was estimated with genotyping quality above 98 % using GCTA software (version 1.94) (Yang et al., 2011). Breeding values were then obtained for each of the accessions using the --reml-pred-rand option from GCTA. This function estimates the variance of a trait explained by all analyzed SNPs. To estimate the weight of each SNP (SNP coefficients) separately, the --blup-snp function from GCTA was used

**Fig. 2.**
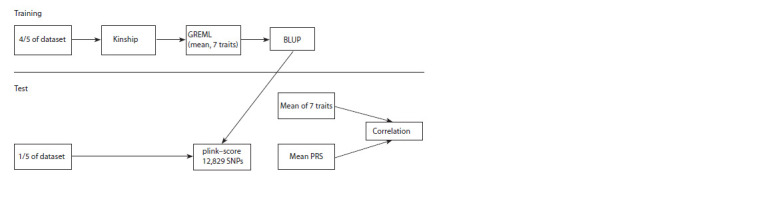
Analysis flowchart using the k-block cross-validation method.

To check the validity of the obtained results, we applied a model using the k-fold cross-validation method (Fig. 2).

The sample of 149 units was randomly divided into five subsamples. Each of the subsamples acted once as a test population, while the remaining four subsamples acted as a training population. Thus, for each of the chemical elements, five models were used to obtain SNP coefficients to assess the breeding value of the element content in wheat. In each case, there were 119 varieties in the training set, and 30 varieties in the test set

Breeding value assessment was carried out using GCTA software as described above. Next, the coefficients of single nucleotide polymorphisms were obtained to assess the breeding value of the content of elements in wheat using the BLUP method implemented in the GCTA software.

For the obtained coefficients, the BV was estimated for all accessions from the test population using plink software (version 1.90b6.26) (Purcell et al., 2007). The obtained values were used to assess the quality of the prediction by calculating the correlation coefficients between the estimated BV and the actual phenotypic data. Confidence intervals for the values of correlation coefficients were also estimated using the Fisher z-transformation of the distribution

For visualization, we used R programming language (version 2022.07.0, build 548). The regression line was constructed using the formula BV ~ phenotype, where BV is the estimated breeding value, and phenotype is the real values of the phenotypes

## Results

**Fig. 3.**
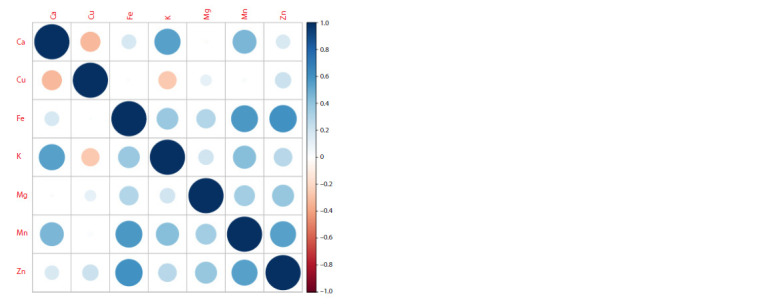
Plot of correlations between phenotypes for 149 varieties. For each variety, the phenotype value was calculated as the average between the four points.

Table 1 and Table 2 show the phenotypic mean and the heritability of each element in 2018 and 2019, respectively. A graph of correlations between phenotypic means is presented in Figure 3

**Table 1.**
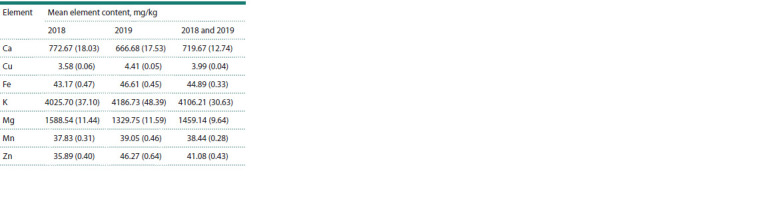
Phenotypic mean values for each year separately (2018 and 2019) and averaged between years, the standard error is indicated in parentheses

**Table 2.**
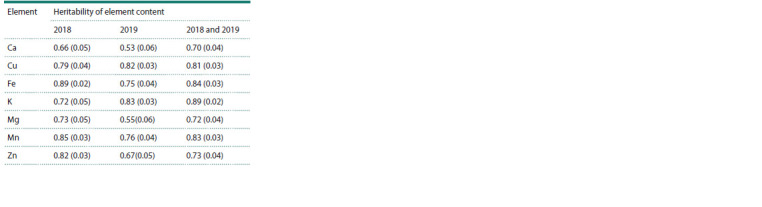
Heritability of traits by year (in fractions of one, where zero is the absence of a genetic contribution to the trait, one is a completely genetically determined trait) and for the average between years, the standard error is indicated in parentheses

Data on estimates of BV and mean content of seven elements for each variety are presented in Supplementary Material 11


Supplementary Materials are available in the online version of the paper: https://vavilov.elpub.ru/jour/manager/files/Suppl_Potapova_Engl_28_4.pdf


Data on the estimated correlation coefficients and confidence intervals, as well as the p-value between the estimated BV and the actual phenotypic data for all seven elements studied, are presented in Supplementary Material 2.

Average values of Pearson correlation coefficients were obtained to predict the concentration of microelements with real phenotypes: K – 0.67, Ca – 0.61, Mg – 0.4, Mn – 0.5, Fe – 0.38, Zn – 0.46, Cu – 0.48. The maximum correlation coefficient was 0.75 ( p-value = 1.85e-07) and was obtained for model 4 for potassium. The minimum is 0.22 for model 5 for iron ( p-value = 0.24).

It was assumed that the prediction of BV for an element is significant if for at least one out of the five models the p-value is below the threshold adjusted for multiple testing ( p-value < 0.001), and for the remaining four models the p- va-lue is below the nominal level of significance ( p-value < 0.05). Thus, we obtained significant estimates of the BV for calcium, potassium and manganese.

The absolute values of the correlation coefficient for the other four micro- and macroelements (Fe, Mg, Zn, Cu) and models were included in the estimated confidence intervals of each model for each of the studied elements, and were also significantly different from zero for 28 out of the 35 estimated models. For iron, in three out of the five models (models numbered 1, 4, 5), the p-values were above the nominal significance level of 0.05. Also, correlation coefficient values insignificant at the p-value level were obtained for model 3 for copper, model 2 for magnesium, model 4 for manganese and model 1 for zinc. The resulting scatterplots are presented in Supplementary Materials 3–9.

For the 30 varieties with the highest estimated BV, the response to selection was assessed (compared with the average values of BV for the population) (Table 3). A comparison was carried out for 30 varieties with the highest values of microand macroelements. Response to selection for phenotypes was adjusted for heritability. Only for calcium, the response to selection obtained while accounting for the BV was higher than the response to selection obtained for phenotypes while taking into account heritability (Table 3). The response to selection was estimated as (Ptop – Pmean)*h2, where Ptop is the average value of the phenotype for 30 varieties with the highest value of the estimated BV, Pmean is the average value of the phenotype in the study population, h2 is the heritability indicator of this phenotype.

**Table 3.**
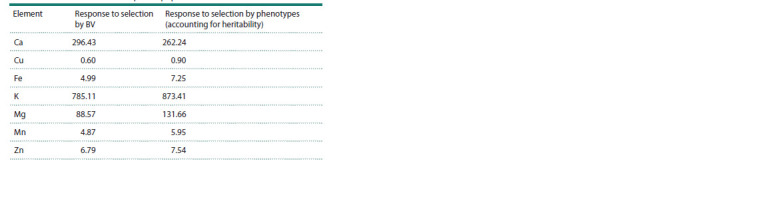
Expected response to selection using 30 cultivars with the highest estimated BV and 30 cultivars with the highest content of micro- and macronutrients as parent population

The resulting estimates of the breeding value of Russian wheat varieties in the form of coefficients for SNP were registered in the Unified Register of Russian Programs for Electronic Computers and Databases and are available upon request to the copyright holder (Institute of Cytology and Genetics SB RAS) (Supplementary Material 3).

## Discussion

In this work, we conducted a study of unbiased estimates of the effects of genetic polymorphisms and their use to assess the genomic potential of Russian spring bread wheat samples for the content of seven micro- and macroelements – K, Ca, Mg, Mn, Fe, Zn, Cu. The best linear unbiased prediction (BLUP) was chosen as a method, and an approach of dividing the sample into several parts (k-fold cross-validation) was selected to check the quality of the model. The choice of model and method was due to the wide dissemination and application of them in genomic selection of plants and animals (Piepho et al., 2008; Molenaar et al., 2018; Tajalifar, Rasooli, 2022).

The sample was randomly divided into five subsamples. Correlation was used as a quality metric for the obtained SNP coefficients to assess the BV. The minimum correlation coefficient value was 0.22 for model 5 for iron content ( p- value = 0.24). At the same time, the p-values of model 5 for all elements were higher than the nominal value of 0.05 for iron in only one case out of seven. Moreover, out of 35 p-values obtained for the correlation coefficients of the estimated BV and real phenotypes, only 7 were equal to or above the nominal significance level of 0.05. This indicates a stable estimate of BV between different parts of the sample.

It is worth noting that for calcium, potassium, and magnesium, at least one out of the five models had a correlation coefficient that was significant using the threshold adjusted for multiple testing ( p-value < 0.001), and the remaining models were significant using the nominal significance level ( p-value < 0.05). Based on this, we established that the estimates of breeding value for these three elements are significant. The lack of significance according to a given criterion for the remaining four elements can be observed due to many factorssuch as small sample size, heterogeneity of the selected population according to the estimated BV, etc. We also measured confidence intervals for each obtained value of the correlation coefficient. For each of the seven elements studied, all correlation coefficient values for all models were within the estimated confidence intervals. 

One of the advantages of using genomic selection, and using BLUP in particular, is the ability to evaluate the expected increase in a trait in the next generation (response to selection). We assessed breeding differentials and response to selection for 30 varieties with the highest BV values and 30 varieties with the highest content of micro- and macroelements in wheat. In the case of selection based on BV the expected response to selection is comparable to the expected response to selection based on phenotypes. This statement is appliable in the case when the response to selection based on phenotypes is weighted by the heritability of the trait according to the breeder’s equation. In our study we showed higher response for selection based on BV for calcium. The obtained high values of the selection differential for selection by phenotypes may be associated with high heritability and heterogeneity in the distribution of phenotypes in the studied population

Previously, we conducted a genome-wide association study for seven micro- and macroelements in varieties and introgression lines of wheat (Potapova et al., 2023), and identified four significant loci. One of them was associated with the content of potassium and calcium, two with the content of iron and manganese, and one with all the studied elements. The results of this work demonstrate that, indeed, by using data from wheat accessions, it is possible to obtain estimated BV numbers for predicting calcium and potassium content (for calcium and potassium, all p-values obtained were less than the nominally significant threshold of 0.05). However, for three out of five models for iron and one out of five for manganese, the p-values exceeded the nominally significant threshold. This may be due to a limited sample size or many other factors, such as the complex genetic structure of a trait (for example, polygenicity or pleiotropy), insufficient data for prediction (number of varieties or SNPs), etc. At this time, there is a lack of scientific publications analyzing the breeding value of varieties for the content of the elements we studied. In this regard, it is difficult to compare our results with previous ones.

The main limitation of this work is a relatively small sample size. There are currently no reliable estimates of what minimum sample size is needed to create genomic selection models. In this article, we empirically showed that it makes sense to carry out such studies even on small samples (149 varieties with four measurements for each, a total of 596 phenotypic points). It is expected that as the sample size increases, the quality of the models will also increase. The second limitation of our work is the use of microarray genotyping data to construct models. If the genotyping test data are obtained using another array or technology, the model we used will most likely be inapplicable due to low overlap in polymorphisms. The use of genetic imputation methods can potentially solve this problem and increase genotyping coverage (Nyine et al., 2019; Song et al., 2019; Munyengwa et al., 2021; Bonnett et al., 2022; Kriaridou et al., 2023), and testing these methods on wheat is the scope of future work in this direction

## Conclusion

Thus, in this work, estimates of the BV were obtained for Russian wheat varieties, regarding the content of seven chemical elements in the grain (K, Ca, Mg, Mn, Fe, Zn, Cu). Our results can be useful primarily for breeders when carrying out work on the selection and breeding of varieties with a high content of micro- and macroelements in the grain. Using the values of the estimated BV, it becomes possible to rank and select the best samples from the populations under study. Additionally, this work can be methodologically useful in creating models for genomic selection of other agricultural plants. Also, these assessments can be used in practice developing breeding schemes and directly breeding new varieties.

## Conflict of interest

The authors declare no conflict of interest.
